# Evaluating the success of Iran Electronic Health Record System (SEPAS) based on the DeLone and McLean model: a cross-sectional descriptive study

**DOI:** 10.1186/s12911-023-02100-y

**Published:** 2023-01-17

**Authors:** Azadeh Bashiri, Mohammad Shirdeli, Fatemeh Niknam, Soheila Naderi, Sahar Zare

**Affiliations:** 1 Department of Health Information Management, School of Health Management and Information Sciences, Health Human Resources Research Center, Clinical Education Research Center, Shiraz University of Medical Sciences, Shiraz, Iran; 2 Department of Health Information Management, Student Research Committee, Health Human Resources Research Center, Shiraz University of Medical Sciences, Shiraz, Iran; 3 Department of Biostatistics, School of Medicine, Shiraz University of Medical Sciences, Shiraz, Iran; 4 Health Information Management Research Center (HIMRC), Kashan University of Medical Sciences, 5th of Qotb -e Ravandi Blvd, Kashan, 87159-73449 Iran

**Keywords:** Electronic health record, Success model, DeLone and McLean

## Abstract

**Background:**

Quality dimensions are the most important criteria for predicting the success of an information system. The current study aims to evaluate the success of the Iran Electronic Health Record System (SEPAS) based on the DeLone and McLean model for information system success.

**Method:**

This nationwide cross-sectional study was conducted in 2021. Participants were 468 health information management personnel who had working experience with SEPAS. Data were collected using a questionnaire based on the DeLone and McLean model. The validity and reliability of the questionnaire were confirmed. Data were analyzed using SPSS 22 through descriptive and analytic analysis including t-test and ANOVA.

**Results:**

Most participants were female (70.9%) and almost half of the participants mean age was between 30 and 40 years old (49.6%). The total mean of SEPAS success was 3.42 ± 0.53. According to the participants’ perspectives “system quality” was the most influencing factor on SEPAS success. The least influencing factor was SEPAS “benefits”. There was a significant relationship between the mean score of SEPAS success and age (*p* value = 0.001), Education level (*p* value = 0.01), and Work experience (*p* value < 0.001).

**Conclusion:**

The total mean of system success was not acceptable. SEPAS has not been much successful in providing net benefits like provision of electronic services which locate patients in the center and improve the delivery of care to them. It sounds that SEPAS is not stable enough that means crashes sometimes. Hence, considering the required infrastructures for quick response and stability is more critical, especially when healthcare providers are supposed to use the SEPAS.

## Background

Electronic Health Record (EHR) are health records residing in an electronic system specifically designed for data collection, storage, and manipulation, and to provide safe access to complete data about patients [1]. EHRs systematically collect lifelong health information about patients’ inpatient, outpatient, and emergency encounters such as medical history, physician orders, nurse notes, vital signs, and Para-clinical investigations.

Considerable capital has been invested in the implementation of EHRs in many developed countries in previous decades [2, 3]. Developing countries, also have taken some steps to employ information technologies including EHR in their healthcare centers. In Iran, efforts to develop infrastructures for and implement EHRs have been part of the Health Ministry IT strategies [4]. Formally, the Ministry of Health in Iran launched a national project as an electronic health records system (SEPAS) in order to develop a national network of health information in 2007. SEPAS is a national electronic health record for all Iranian citizens that collects patients’ data on each patient encounters from all hospitals in a centralized system [5]. Also, the Patients’ data including demographic information, final diagnosis, procedures, para-clinical results, and accounting billing from hospitals’ information systems (HIS) are transferred to SEPAS and stored in the Ministry of Health databases to be used in the future. The software infrastructure instances are installed in several locations as SEPAS nodes, hosted by each medical university in the country. Hospitals which are under the supervision of the associated medical universities exchange health data through their corresponding SEPAS nodes [6, 7]. Management Centre of Statistics and Information Technology of the ministry is accountable for its development. Now, after about 15 years since 2008, Iran has moved from a starting point of no EHR state to a national wide coverage; with a large amount of patients’ information communicated to and stored in SEPAS as a data warehouse for comprehensive implementation of national EHR. However, it is not clear whether this project was successful enough.

The barriers to EHR successful implementation include user resistance, lack of skills, lack of engagement of frontline clinicians, concern for return on investment, lack of administrative and policy support, and the longer time of using EHR in comparison to paper-based [8–10]. Thus, to implement successful comprehensive EHR, prior evaluations are necessary. Similar studies evaluating the success of EHR indicated a need for improving the EHR [11–13]. Few studies have been conducted for the comprehensive evaluations of EHRs in Iran. Asadi et al. [14] evaluated the SEPAS project and found that the required resources and requirements have not been considered for the project and consequently it has not been able to meet the pre-defined objectives. However, the viewpoints on the success of EHRs have not been extensively sought; while any shortcoming can negatively affect the usage decisions. Thus, there is a gap in the literature in regard to the evaluation of SEPAS in hospitals. Considering the increasing importance of the SEPAS system in Iran and the capital invested in its development by the Ministry of Health, assessment of the success of SEPAS is essential to understand the value and effectiveness of this information system and justify the capital invested in its development and implementation. Thus, the current study aims to evaluate success of Iran EHR system (SEPAS) based on the DeLone and McLean model for information system success.

## Method

### Study design and setting

This nationwide cross-sectional study was conducted in 2021. Iran includes 31 provinces with 570 public hospitals including 271 teaching hospitals. Only teaching hospitals were included in this study. Convenient sampling was used to select hospitals from some provinces including Fars, Tehran, Khoozestan, Khorasan Razavi, north khorasan, Hamedan, Isfahan, Chaharmahal va Bakhtiari, and Yazd.

### Participants

The target population included healthcare employees working with SEPAS. Patients’ information is transferred from HISs to the SEPAS by the healthcare workers especially health personnel in health information management department and personnel in accounting department of the hospitals. SEPAS is now used to store healthcare information of the patients’ encountering hospitals and is not used by healthcare providers including physicians and nurses. Thus, our target population was health information management personnel who had working experience with SEPAS. The sample size was calculated 468 using Gpower software; to estimate the “system success” score, sample size was calculated based on the effect size obtained from [15] which was 0.13. Significance level, power, and accuracy of estimation were considered 0.05, 0.80, and 0.1 respectively. We used Convenience sampling technique in which the subjects are selected based on availability and willingness to participate.

### Data collection

**Fig. 1:**
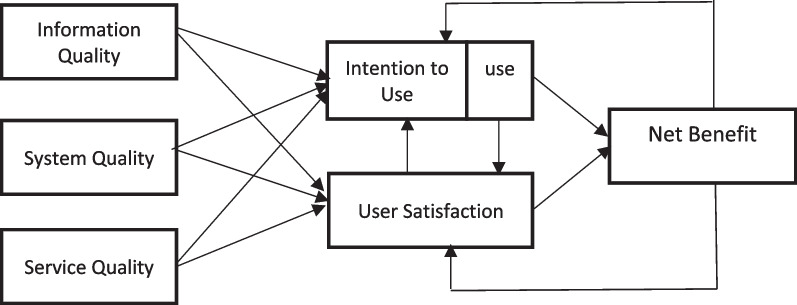
Updated DeLone and McLean information system model

Data were collected using a questionnaire based on the DeLone and McLean model, one of the popular and most validated models for information system success [16, 17]. The model proposes six interrelated constructs of information systems success including system quality, information quality, service quality, (intention to) use, user satisfaction, and net benefits (Fig. 1). Information quality is defined as the quality of the information provided by the system (system output). System quality refers to the desirable features/the overall support that a system provides. Intention to use/use is defined as the users’ intention to use or the perceived actual usage of an information system by users to accomplish multiple tasks. User satisfaction refers to the users’ level of satisfaction when using an information system. Net benefits refer to the extent to which an information system contributes to the individuals, organizations, and group success. These variables measure technical success, semantic success, and effectiveness success of an information system. The questionnaire was developed based on the questionnaire used in [11, 17, 18]. Four experts in health information management with at least ten years’ experience in the field confirmed the content validity of the questionnaire after some revisions. The overall reliability of the questionnaire was calculated through Cronbach’s alpha (á = 0.862). Final questionnaire was consisted of 27 questions categorized in six dimensions including system quality (five questions), information quality (six equations), services quality (six equations), (intention to) use (four equations), user satisfaction (two equations), and net benefits (four equations). Each question is rated based on a five-point Likert score from 1 (disagree) to 5 (agree). The questionnaire was distributed face to face by one researcher, or through online questionnaire link via social media groups consisting target members. Distributing questionnaires continued till the targeted sample size was obtained; this process lasted for about 4 months.

### Data analysis

Data were analyzed using SPSS.22. Descriptive statistics were used to report the frequency, percentages, and mean for describing the participants’ characteristics as well as SEPAS success dimensions. T-test was used to compare the rate of SEPAS success according to gender; the ANOVA test was used to examine the rate of SEPAS success according to age groups, educational level, and work experience. Where the ANOVA test showed insignificant differences between groups’ variances, the LSD test was used to make pairwise comparisons between groups, and where the ANOVA test showed significant differences between groups’ variances Tamhane’s T2 test was used for pairwise comparisons. The mean score and standard deviation (SD) are used to describe SEPAS success. Since the number of items in each construct is different, to make constructs comparable, we also used this formula [(mean of construct/5) * (100)] to compute the scores out of 100. The total score of SEPAS success was calculated based on the averages of total scores of constructs out of 100. To determine whether the level of SEPAS success based on the quality of each six constructs is acceptable or not, a one-way t-test was used. If at least 75% score (3.75 out of 5) is obtained for each item, the status will be considered acceptable [15].

### Ethical consideration

The research is conducted according to the principles stated by the Vice-Chancellorship for Research Affairs of Shiraz University of Medical Science and is approved by the Ethics Review Board of the Vice-Chancellorship for Research Affairs of Shiraz University of Medical Science (Ethical code: IR.SUMS.REC.1399.798).

## Results

468 respondents participated in the study. Demographic data of the healthcare employees showed that most of the participants were female (70.9%) and almost half of the participants’ mean age was between 30 and 40 years old (49.6%). Most of the participants had a “bachelor’s science” degree (69%). Moreover, the work experience of most participants (43.6%) in the health information management department was more than ten years (Table 1).

**Table 1:** Demographic data of the participants’ students

Characteristics	Number*	Percent
*Gender*		
Female	332	70.9
Male	136	29.1
Age		
< 30	59	12.6
30 ≤ x < 40	232	49.6
40 ≤ x < 50	144	30.8
≥ 50	33	7.1
*Education level*		
Associate science	55	11.8
Bachelor science	320	68.4
Master or higher	93	19.9
*Work experience*		
1 ≤ x < 5	99	21.2
5 ≤ x < 10	165	35.3
≥ 10	204	43.6

*Total n = 468

Table 2 shows the descriptive statistics of SEPAS success constructs. The total mean of SEPAS success was 3.34 ± 0.37 (61.9 out of 100). According to the participants’ perspective “system quality” obtained the most score (71.8 out of 100). “Benefits” obtained the least score (60.05 out of 100). In the system quality construct, the highest and the lowest means were regarded as “It has been easy for me to learn how to use SEPAS” (4.04 ± 0.76), and “SEPAS is stable to a satisfactory degree and crashes seldom” (2.78 ± 0.94) respectively. The participants were satisfied with using SEPAS (3.46 ± 0.74). The participants believed that using SEPAS is not much beneficial and based on their viewpoints the most beneficial feature of SEPAS is “improving decision making” (3.33 ± 0.92) and the least beneficial feature was “improving patient care delivery” (2.80 ± 1.04). Among all items “It has been easy for me to learn how to use SEPAS” was the most influencing factor causing SEPAS success while “It allows me to provide feedback about the system and its services” was the least influencing factor.

**Table 2:** descriptive statistics of SEPAS success constructs

SEPAS success subscales	Mean	SD	Total score out of 100
1. Information are accessible	3.62	0.86	72.4
2. Information appears orderly and easy to read	3.28	0.85	65.6
3. It is easy to review information	3.56	0.80	71.2
4. Information are always updated	3.53	0.85	70.6
5. It provides information required for work	3.17	0.83	63.4
6. It is easy to document information in the right places	3.64	0.74	72.8
Information quality	3.47	0.51	69.3
7. Its response time for login is satisfactory	3.96	0.76	79.2
8. It is easy to learn how to use SEPAS	4.04	0.76	80.8
9. It is easy transactions	3.83	0.89	76.6
10. It responds rapidly and satisfactorily when shifting between screens	3.34	0.81	66.8
11. It is stable to a satisfactory degree	2.78	0.94	55.6
System quality	3.59	0.46	71.8
12. I am satisfied with the support I received	3.39	1.0	67.8
13. It is easy communication with the support team	3.05	0.90	61
14. I am satisfied with the security and privacy policies	3.72	0.74	74.4
15. It is clearly stated security and privacy policies	3.10	0.88	62
16. It allows me to provide feedback about the system and its services	2.75	0.97	55
17. I am satisfied with the available user guides and help functions	3.04	0.96	60.8
Services quality	3.17	0.51	63.5
18.Implementation of SEPAS has improves work procedures	3.61	0.97	72.2
19. Implementation of SEPAS entails new tasks	4.11	0.76	82.2
20. Implementation of SEPAS has meant that I have handed over tasks to others	2.77	0.90	55.4
21. SEPAS has replaced paper records, but has also entailed new documentation on paper	3.01	1.74	60.2
Use	3.38	0.62	67.5
22. Generally, SEPAS has made my work easier	3.24	0.89	64.8
23. I am satisfied with SEPAS	3.68	0.93	73.6
Satisfaction	3.46	0.74	69.2
24. SEPAS helps overcome the limitations of the paper-based system	3.06	1.09	61.2
25. Using the SEPAS will cause an improvement in patient care delivery	2.80	1.04	56
26. SEPAS will enhance communication among workers	2.82	0.87	56.4
27. SEPAS use will cause improved decision making	3.33	0.92	66.6
Benefits	3.00	0.61	60.05
SEPAS success	3.34	0.37	61.9

1 = Disagree; 2 = Disagree somewhat; 3 = Neither disagree nor agree; 4 = Agree somewhat; 5 = Agree

Investigating the correlations between the mean score of SEPAS success and demographic characteristics of the employees showed that there were significant correlations between the mean score of SEPAS success and gender (0.007), age (*p* value = 0.001), Education level (*p* value = 0.023), and Work experience (*p* value < 0.014). Leven test confirmed that there were homogenous variances between age groups (*p* value = 0.466). Accordingly, multiple comparisons using the LSD test showed that the mean score of SEPAS success was less in participants < 30 years old. Likewise, the results of the LSD test (Levene *p* value = 0.104) showed a significant difference between participants with a bachelor’s science degree and those with a master’s science degree, between the mean score of SEPAS success was less in those with master’s or higher degree. There was also a significant difference between work experience 1–5 and work experience ≥ 10 (*p* value = 0.014) (Table 3).

**Table 3:** Mean score of SEPAS success based on the participants’ demographic information

Characteristics	Mean (SD)	*p* value
*Gender*		
Female	3.37 (0.38)	0.007
Male	3.27 (0.35)	
Age		
< 30	3.17 (0.39)	< 0.001
30 ≤ x < 40	3.34 (0.39)	
40 ≤ x < 50	3.413(0.32)	
≥ 50	3.40 (0.29)	
*Education level*		
Associate science	3.35 (0.27)	0.023
Bachelor science	3.37 (0.39)	
Master or higher	3.25 (0.36)	
*Work experience*		
1 ≤ x < 5	3.26 (0.42)	0.014
5 ≤ x < 10	3.34 (0.33)	
≥ 10	3.39 (0.37)	

One sample t-test results are shown in Table 4. Considering *p* = 0.00, the assumption (H: μ > 3.75) would be rejected. This demonstes that  the SEPAS success rate is not acceptable which means at least a 75% score (3.75 out of 5) is not obtained for any of the items.

**Table 4:** the rate of SEPAS success based on quality of the six constructs

Constructs	Mean	SD	*p* value
Information quality	3.470	0.512	< 0.001
System quality	3.598	0.465	< 0.001
Service quality	3.179	0.514	< 0.001
Use	3.380	0.620	< 0.001
Satisfaction	3.464	0.744	< 0.001
Benefits	3.008	0.617	< 0.001

## Discussion

The current national study evaluated the Iran EHR system (SEPAS) based on the DeLone and McLean model for information system success. The respondents believed that the system was not successfully implemented. Although the participants believed that the “System quality” was almost well enough, implementing SEPAS does not sound “beneficial”. Moreover, employees with master’s or higher degrees believed that the SEPAS system was less successful in comparison to those with bachelor’s science degrees. “Ease of using the system” was the most influencing factor causing SEPAS success.

Generally, the health information management personnel hold positive views on how the quality of the SEPAS and the quality of its information. They believed that SEPAS has the required functionality to support the work in question and is easy to use. These results are consistent with the previous studies [19–22]. For instance, the participants in a study [20] believed that the EMR system provides the required information about patients and they were satisfied with the accuracy of the system. Moreover, another study [22] found that the respondents were largely positive about the quality of the EHR and were satisfied with using it. Similarly, Saghaeiannejad et al.’s [23] study showed that “ease of learning and use” was the most desirable factor according to the users’ responses. However, it sounds like SEPAS is not stable enough which means crashes sometimes. In order to improve system quality considering the required infrastructures is necessary before EHR implementation. The users in Saghaeiannejad et al. [23] research also believed that the “response time” of the system was not desirable. Researchers believe that continuously upgrading and optimizing the IT infrastructure including computers, cables, and the wireless network would probably contribute to good system quality [11]. On the other hand, the SEPAS infrastructures, are guaranteed to provide high bandwidth and secure communication channels. This secure channel is provided using the SHAMS network, a national private health information network. Transferring health data using the SHAMS network guarantees the confidentiality of patients’ data [7]. SEPAS is not used by physicians or nurses currently, but when healthcare providers are supposed to use SEPAS, quick response and stability are more critical. Spending time using EHRs for supporting care delivery constitutes a large portion of physicians’ day [24]. Despite the increased adoption of EHRs, concerns about the adverse consequences of EHRs use on healthcare providers’ satisfaction and burnout are growing [25]. These concerns necessitate developing EHR innovations. Some of these innovations provide automatic data input [26]. SEPAS is now fed through hospital information systems and is not integrated with any other devices including mobile documentation applications. Nowadays, EHR innovations necessitate integrating EHRs with many devices which led to decreased click burden and allows clinicians to spend less time on documentation and more time with patients [26]. On the other hand, these innovations may cause even more crashes if the required infrastructures are not considered in advance.

The respondents reported that SEPAS benefits are almost low in regard to removing paper-based systems limitations, improving patient care, or improving communication among healthcare workers. Bossen et al. [11] found that healthcare staff had a very high expectation of future benefits before full implementation of EHR. Perceived net benefits of systems ultimately drive system success [17]; thus, if users do not feel system benefits may reduce its adoption. According to the health information management personnel, SEPAS has not been much successful in providing electronic services which locate patients in the center and improve the delivery of care to them.

Results also showed that educational level influence the perceived system success. Employees with master's or higher degrees believed that the SEPAS system was less successful in comparison to those with bachelor’s science degrees. This finding is inconsistent with similar researches [27, 28] who indicated that educational level is a technology adoption moderator, the higher the education level the higher is the technology adoption. An explanation for this inconsistency may be the more knowledge of employees with a higher degree about information technologies which has influenced their expectation about an optimal information system.

Although the total mean of system success was in the acceptable range, it was far from the ideal situation (highest score). This is consistent with the Saghaeiannejad et al. [23] findings. Thus, it is important to develop EHRs based on end users’ requirements and expectations rather than the developers’ expectations. The Iran EHR implementation plans at the national level seem ambitious in comparison to other countries; however, it is important to pay attention to the required communication infrastructures, digital health data regulations, and communication standards. Healthcare centers are suggested to implement a local EHR according to their organizational systems, process, and workflows rather than trying to develop a national EHRs which are difficult to integrate with the existing systems. The integration and communication of these local EHRs can be facilitated through the development of communication standards that each healthcare center would comply with.

A limitation of this study is that the assessment was based on the health information management staff, while financial staffs are also the users of the SEPAS whose viewpoints were not investigated. Since financial departments’ staffs have the responsibility of entering data through a similar user interface; it was supposed that they may have similar viewpoints to health information management staffs. Viewpoints of financial departments’ staffs can be addressed in future studies.

## Conclusion

We have presented a formative evaluation of a national EHR (SEPAS) based on health information management personnel’s point of view. The total mean of system success was not acceptable. SEPAS has not been much successful in providing net benefits like the provision of electronic services which locate patients in the center and improve the delivery of care to them. It sounds like SEPAS is not stable enough which means crashes sometimes. Hence, considering the required infrastructures for quick response and stability is more critical, especially when healthcare providers are supposed to use the SEPAS.

## Data Availability

All data will be presented upon request via contacting the corresponding author.

## Abbreviations

SEPAS: Iranian electronic health record (translation of the Persian phrase)
EHR: Electronic health record
EMR: Electronic medical record
